# Cytotoxic Effects of “25.2% Boscalid + 12.8% Pyraclostrobin” Fungicide

**DOI:** 10.1002/jat.4864

**Published:** 2025-07-26

**Authors:** Yasin Eren

**Affiliations:** ^1^ Department of Pathology Laboratory Techniques Şuhut Vocational School of Health Services, A fyonkarahisar Health Sciences University Afyonkarahisar Turkey

**Keywords:** 25.2% boscalid‐12.8% pyraclostrobin, fungucide, MTT assay, toxicity, Triticum test

## Abstract

It is known that different pesticides used against domestic or agricultural pests have toxic effects. In this study, 25.2% boscalid and 12.8% pyraclostrobin were used in the test material. Kate A1 Russian wheat variety was used for Triticum growth inhibition tests. According to the Triticum root and stem growth test, the concentration value that halves the root and stem length is known as the “EC50 value” According to the test, the root length of the control group was 6.98 ± 0.65 cm and the length of the stem was 9.36 ± 0.71 cm. According to the Triticum test, the EC50 value of the fungicide was determined as 2500 ppm. The value that halves the stem length of the control group was determined as 1250 ppm. Some doses of this fungicide (625, 1250, 2500, 5000, and 10,000 ppm) were observed to inhibit root and stem growth, and these concentrations' results were statistically significant according to Dunnett's *t*‐test. In the root mitotic index analysis, 5000 cells were counted for each concentration, and it was determined that all concentrations tested had negative effects on mitotic activity. It was observed that the concentration of 10,000 ppm was the most decreasing (0.53 ± 0.18 cm) mitotic index %. The recommended dose of the tested fungicide in the fight against agricultural pests is around 500 ppm, and this indicates that the fungicide will have limited cytogenetic effects. The mitotic index test indicated that 2500 and upper concentrations had toxic effects on the mitotic index. The most toxic effect was in the 10,000 ppm treatment. The MTT test showed that all concentrations had a cytotoxic effect on MDBK cells. But 625 ppm concentration in all treatment periods and 1250 ppm 24‐h results were statistically no significant according to Dunnett's *t*‐test.

## Abstract 2

Approximately 25.2% boscalid and 12.8% pyraclostrobin fungicide is one of the pesticides that have been increasingly used in recent years to control pistachio, olive, and pomegranate pests. According to the Triticum test, EC50 value of fungicide was determined as 2500 ppm. Mitotic index test indicated that 2500 and upper concentrations had toxic effects on mitotis. MTT test showed that all concentrations had cytotoxic effect on MDBK cells.

## 1 Introduction

The boscalid‐based fungicides Endura (70% boscalid) and Pristine (25.2% boscalid and 12.8% pyraclostrobin) were registered in the United States in 2003 (Landschoot et al. 2017). Fungicides are a type of pesticide that is frequently used in agricultural lands to control pathogenic fungi on plants. Fungicides are used to treat and preserve maize, wheat, olive, pistachio, and fruit, as well as other plants (Chen et al. 2008; Avenot et al. 2008; Temiz 2019). The 25.2% boscalid and 12.8% pyraclostrobin fungicide is one of the pesticides that have been increasingly used in recent years in Turkey to control pistachio, olive, and pomegranate pests. Boscalid is a novel broad‐spectrum fungicide that belongs to the anilide family of fungicides. Its action mode and range are different from those of strobilurins and most of the other fungicides. Boscalid inhibits complex II in the mitochondrial electron transport chain, whereas pyraclostrobin inhibits complex III (Avenot et al. 2008; Lagunas‐Allué et al. 2015; Ozkılınc and Kurt 2017; Aksakal 2020). There is emerging correlative and epidemiological evidence that at least some fungicides can be harmful to bee health (McArt et al. 2017).

The 25.2% boscalid + 12.8% pyraclostrobin fungicide is very toxic to fish, to aquatic invertebrates, and acutely toxic for aquatic plants (OECD Guideline 2022). LC50 (96 h) value is 0.042 mg/L for *Oncorhynchus mykiss* (OECD Guideline 203, 2022), EC50 (48 h) value is 0.08 mg/L for *Daphnia magna* (OECD Guideline 202, 2022), and EC50 (72 h) value is 4.99 mg/L (growth rate) for *Pseudokirchneriella subcapitata* aquatic plant (OECD Guideline 201, 2022).

This fungicide is slightly toxic after single ingestion, slightly toxic after short‐term skin contact, and relatively nontoxic after short‐term inhalation. Oral toxicity for rat is LD50 1490 mg/kg, LC50 for rat > 5.4 mg/L with inhalation and no mortality was observed with inhalation in 4 h exposure period, and LD50 value for dermal toxicity is > 2000 mg/kg (OECD Guideline 2022).

## 2 Materials and Methods

The 25.2% boscalid + 12.8% pyraclostrobin fungicide was purchased from BASF Turk. Triticum tests were carried out with Kate A1 Russian wheat and different concentrations of the 25.2% boscalid + 12.8% pyraclostrobin fungicide (625, 1250, 2500, 5000, and 10,000 ppm) that were used for the root and stem growth inhibition test.

### 2.1 Root and Stem Growth Inhibition Test (EC_50_ Determination)

Various concentrations of the 25.2% boscalid + 12.8% pyraclostrobin (625, 1250, 2500, 5000, and 10,000 ppm) were used for the root and stem growth inhibition test. The wheats were grown in freshly made distilled water for 24 h and then exposed for 96 h to the control group and other concentrations of 25.2% boscalid + 12.8% pyraclostrobin. In order to determine efficient concentration (EC50) values, 10 roots from each wheat were cut off at the end of the treatment period, and the root and stem's lengths were measured. The concentration that decreased root growth about 50% when compared to the negative control group (distilled water) was accepted as the EC50 value.

### 2.2 Mitotic Index (MI) Determination

At the end of 72 h, root tips were cut and fixed in ethanol: glacial acetic acid (3:1); they were hydrolyzed in 1 N HCl at 60°C for 7 min. Root tips from each concentration treatment were stained with Feulgen dye for 1 h. Five slides were prepared for each concentration, and 1000 cells per slide were counted. A total of 5000 cells were evaluated for each concentration. In the MI study, about 5000 cells were counted, and MI% was determined with the following formulation:

MI%=divided cell number/total cell number×100

### 2.3 MTT Assay

This test was performed with MDBK cells (Madin‐Darby Bovine Kidney) (Sigma) according to Mosmann (1983), and the test was repeated three times. Cells were incubated with different concentrations of fungicide. Then test materials were removed at the end of the incubation period. Cells were incubated with 5 mg/mL MTT solution (Sigma) about 2 h in a CO_2_ incubator for the transformation of MTT dye to formazan salt (not dissolve in water). Then MTT dyes were removed, and 100 μL DMSO was added to the wells in order to dissolve the formazan salts that were only formed by alive cells. Plates were analyzed by ELISA at a 540‐nm wavelength. Cell proliferation of the control group was accepted as “0” (Mosmann 1983).

## 3 Results and Discussion

The boscalid and pyraclostrobin residues were found in some fruit samples (Balkan and Yılmaz 2023). This shows that this fungicide should be considered in terms of human health. It was aimed to carry out this study with this perspective. The findings of the present study demonstrate that the Triticum root elongation assay is a simple and inexpensive method when used empirically for the screening of novel chemical compounds. The MTT (Tetrazolium Blue) colorimetric assay has been reported in previous studies to evaluate the reduction of cell viability in the presence or absence of tested materials (Betancur‐Galvis et al. 1999). It has been stated that cell proliferation can be determined using the MTT assay, which is a useful method for measuring cell viability through mitochondrial dehydrogenase activity (Mosmann 1983).

**FIGURE 1:**
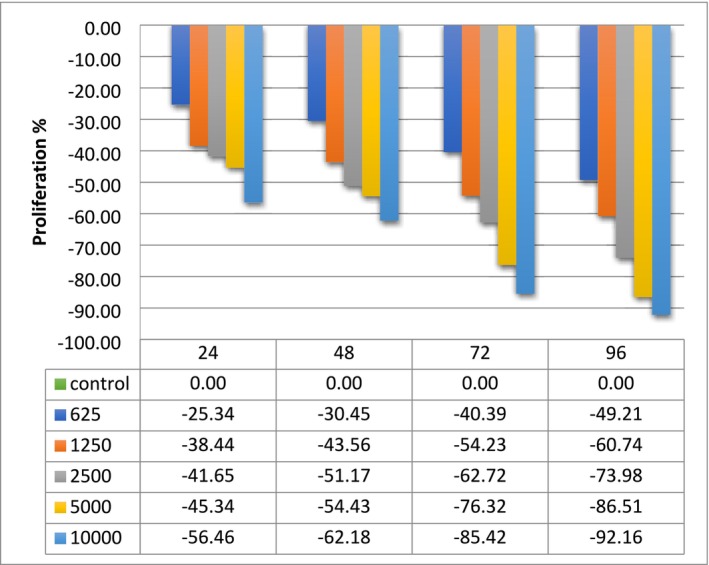
The 25.2% boscalid + 12.8% pyraclostrobin fungicide MTT test results.

Previous study about in vitro unscheduled DNA synthesis (primary rat hepatocytes) indicated negative response up to 50 μg/mL, but cytotoxicity was observed at 100–500 μg/mL. In this study also, gene mutation bacterial reverse mutation assay showed that negative without and with S‐9 activation up to limit dose of 5000 μg/plate. So fungicide combination has positive mutagenic effects in and above 5000 μg/plate (EPA 2003). According to the studies conducted with the Triticum test to determine the MI and stem and root growth test, dose‐dependent decrease was observed. Tested fungicide reduced the MI to max 9.02 cm, and EC50 concentration was found 2500 ppm. However, when compared to the control values, whole of the concentrations were also found to cause toxic effects, except 625 ppm. The toxic effect values are provided in Table 1. The lowest MI was found in 10,000 ppm concentration. According to the MTT test results conducted with the MDBK cell line, it was determined that fungicide exhibited cytotoxic effects at all concentrations except for 625 and 1250 ppm after 24 h of application. When compared to the control group, the most significant negative effect on MDBK cell proliferation was observed with the 10,000 ppm concentration after 96 h of application. It was found that concentrations of 1250 ppm and above have a toxic effect. Figure 1 and Table 2 demonstrated the 25.2% boscalid + 12.8% pyraclostrobin fungicide MTT test results. Although manufacturers recommend a maximum concentration of 1000 ppm for the application of fungicide, the results of this study indicate that when fungicide is applied at high concentrations, cytotoxic effects may be observed on the cells.

**TABLE 1:** Root and stem growth inhibition test results with Triticum test.

Dose	Root length	Stem length	Root mitotic index
Control	6.98 ± 0.65	9.36 ± 0.71	35.6 ± 5.88
625 ppm	6.57 ± 0.84	8.97 ± 1.65	34.52 ± 4.42
1250 ppm	4.17 ± 0.42^a^	4.24 ± 0.63^a^	22.53 ± 2.64^a^
2500 ppm	3.53 ± 0.47^a^	3.23 ± 0.60^a^	19,86 ± 3.02^a^
5000 ppm	3.26 ± 0.40^a^	1.26 ± 0.34^a^	17,32 ± 3.18^a^
10.000 ppm	1.70 ± 0.33^a^	0.53 ± 0.18^a^	9.02 ± 1.85^a^

^a^ Significant according to Dunnett's *t*‐test (*p* < 0.05).

**TABLE 2:** The 25.2% boscalid + 12.8% pyraclostrobin fungicide MTT test results.

Dose	Exposure time			
ppm	24 h	48 h	72 h	96 h
625.00	0.05 ± 0.03	0.07 ± 0.05	0.10 ± 0.09	0.14 ± 0.04
1250.00	0.17 ± 0.08	0.19 ± 0.04^a^	0.26 ± 0.08^a^	0.30 ± 0.07^a^
2500.00	0.22 ± 0.07^a^	0.28 ± 0.10^a^	0.34 ± 0.13^a^	0.41 ± 0.12^a^
5000.00	0.46 ± 0.18^a^	0.50 ± 0.21^a^	0.54 ± 0.23^a^	0.63 ± 0.25^a^
10.000.00	0.62 ± 0.28^a^	0.70 ± 0.31^a^	0.74 ± 0.32^a^	0.82 ± 0.26^a^
Control	0	0	0	0

^a^ Significant according to Dunnett's *t*‐test (*p* < 0.05).

## Conflicts of Interest

The author declares no conflicts of interest.

## Data Availability

The data that support the findings of this study are available from the corresponding author upon reasonable request.
